# TORCH Antibodies Among Pregnant Women and Their Newborns Receiving Care at Kilimanjaro Christian Medical Centre, Moshi, Tanzania

**DOI:** 10.24248/EAHRJ-D-16-00340

**Published:** 2017-07-01

**Authors:** Aliasgher M Saajan, Mramba Nyindo, Joshua G Gidabayda, Mohammed S Abdallah, Shaneabbas H Jaffer, Aliasgher G Mukhtar, Tima M Khatibu, Rune Philemon, Grace D Kinabo, Blandina T Mmbaga

**Affiliations:** a Department of Paediatrics and Child Health, Kilimanjaro Christian Medical Centre, Moshi, Tanzania; b Kilimanjaro Christian Medical University College, Moshi, Tanzania; c Kilimanjaro Clinical Research Institute, Moshi, Tanzania

## Abstract

**Background:**

Toxoplasmosis, other (syphilis, varicella-zoster, parvovirus B19, and hepatitis B), rubella, cytomegalovirus (CMV), and herpes simplex virus type 1 and type 2 (HSV-1 and HSV-2) – known by the acronym TORCH – is a group of infections affecting both mothers and their unborn babies with adverse short- and long-term outcomes. The majority of infected mothers are asymptomatic, which leaves only speculation as to the probable cause of many congenital anomalies, stillbirths, prematurity, and death resulting from TORCH infections. The main objective of this study was to investigate previous exposure to TORCH infections by measuring the seroprevalence of TORCH antibodies in pregnant women and their newborns receiving care at Kilimanjaro Christian Medical Centre (KCMC), Moshi, Tanzania.

**Methods:**

This was a cross-sectional, hospital-based study conducted at KCMC from December 2013 to April 2014. Of 350 pregnant women enrolled in the study, we tested 347 pregnant women attending the antenatal clinic and who opted to deliver at KCMC. Cord blood was collected and analysed for 309 of their newborns. To identify immunoglobulin G (IgG) and immunoglobulin M (IgM) antibodies in mothers and IgM antibodies in newborns, we used enzyme-linked immunosorbent assay testing. A structured questionnaire was used to collect data of mothers and their newborns. Data analysis was done using SPSS version 20.

**Results:**

The seroprevalence of IgG antibodies to TORCH infections among pregnant women was 154 (44.4%) for toxoplasmosis, 311 (89.6%) for rubella, 343 (98.6%) for CMV, and 346 (99.7%) for HSV-1 and HSV-2; 141 (40.6%) had been exposed to all 4 infections. For HSV-1 and HSV-2, the IgM antibodies were found in 137 (39.5%) of the 347 pregnant women included in this study. Age above 35 years (OR 6.15; 95% CI, 1.22–31.1; *P*=.028) and multiparity (OR 1.63; 95% CI, 1.01–2.62; *P*=.045) were associated with higher risk of being exposed to all TORCH infections. A total of 11 newborns had IgM antibodies to HSV-1 and HSV-2 giving a seroprevalence of 3.6%, and one newborn had IgM antibodies to rubella, giving a seroprevalence of 0.3%. None of the newborns had antibodies to toxoplasmosis and CMV.

**Conclusion:**

Exposure to TORCH infections was high among pregnant women in our population. Older age and multi-parity were associated with a higher risk of being exposed to all TORCH infections. Seroprevalence to HSV-1 and HSV-2 was high in newborns. The higher IgM antibodies to HSV-1 and HSV-2 among pregnant mothers and their newborns may disturb maternal, fetal, and neonatal health, and therefore we recommend establishing treatment protocol to support management of pregnant women and newborns who are seropositive for IgM antibodies.

## INTRODUCTION

A wide range of microorganisms including bacteria, viruses, fungi, and protozoans may infect a pregnant woman and can lead to fetal death, organ injury, or short- and long-term sequelae depending on the offending pathogen.^1^ The classical group of microorganisms is known as TORCH, which includes toxoplasmosis, other (parvovirus B19, varicella-zoster virus infection, syphilis, hepatitis B), rubella, cytomegalovirus (CMV), and herpes simplex virus types 1 and 2 (HSV-1 and HSV-2).^2^

These organisms acquired in utero can lead to resorption of the embryo, abortion, stillbirth, malformation, intrauterine growth restriction, prematurity, or sequelae of chronic postnatal infection.^3^ Infection acquired during the intrapartum or early postpartum period may result in severe systemic disease that leads to death or persistent postnatal infection.

In most cases, maternal illness due to TORCH infections is mild, but the impact on the developing fetus is more severe.^4^ Clinical evidence of TORCH infections may be seen at birth, soon afterwards, or not until weeks, months, or years later. This is exemplified by a study of a large cohort of newborns with congenital toxoplasmosis in Brazil, which showed high rates of early retinochoroidal lesions (∼80%) and active lesions (∼50%).^5^ Prevalence of toxoplasmosis based on the detection of immunoglobulin G (IgG) antibodies was reported to be 30.9% in the Mwanza Region of Tanzania.^6^ Worldwide, overall risk of transmission for congenital toxoplasmosis is reported to be 30% and increases with gestational age at maternal seroconversion, from less than 15% at 13 weeks of gestation to almost 71% at 36 weeks of gestation. Prevalence of congenital infection ranges from 0.1 to 0.3 per 1,000 live births.^7^ The classic triad of signs suggestive of congenital toxoplasmosis includes chorioretinitis, hydrocephalus, and intracranial calcifications.^8^

Worldwide, CMV seroprevalence among women of reproductive age ranges from 45% in higher-income countries to 100% in lower- and middle-income countries.^9^ According to a recent review of 11 studies, CMV seroprevalence of adolescents is 90% and >95% during early adulthood and the average transmission rate is 0.65%, ranging from 0.6% in Panama to 6.1% in China.^10^ The same review showed a range of 0%–29% classified as symptomatic CMV at birth.^10^ Another review of 15 studies found that long-term sequelae from congenital CMV occurred 3 to 4 times more in symptomatic infants (40%–58%) compared with asymptomatic infants (13.5%). More children with long-term sequelae from congenital CMV were asymptomatic at birth.^11^

In a study from 2011, CMV had been detected in about 20% of children in daycare centres, but CMV was also detected in about 10% of children who were not in daycare centres.^12^ In a 2017 study in China, a seroprevalence of 96.2% among pregnant women was reported with a CMV transmission rate ranging from 0.4% to 0.7% depending on the specimen screened.^13^ In a study from Japan in 2006, congenital CMV was directly responsible for a substantial proportion of early childhood sensorineural hearing loss, and almost half of the infants at risk for the development of late onset CMV or gap junction beta-2 protein associated sensori-neural hearing loss showed no clinical or audiological indications at birth.^14^

Screening data from the Herpevac Trial for Women revealed that half (51%) of participants screened had antibodies for HSV-1 (with or without HSV-2) and 11% had HSV-2 antibodies with or without HSV-1.^15^ The prevalence of antibodies to HSV-1 and HSV-2 increased with age, between the ages of 18 and 30. In Tanzania, HSV-2 sero-prevalence was 80% among women at high risk in northwestern Tanzania,^16^ 20.7% among pregnant women in the rural Manyara and Singida regions,^17^ and 35% in Dar es Salaam.^18^ Neonatal HSV infection is acquired during 3 distinct periods: intrauterine (in utero 5%), peripartum (during labour and delivery 85%), and postpartum (post-natal 10%).^19^ Intrauterine infection is associated with severe HSV infection regardless of the timing of the acquisition during gestation; at birth, it is characterized by a triad of findings, including skin vesicles or scarring, eye damage, and severe manifestations of microcephaly or hydranencephaly.^20^ Data from the United States showed substantial utilization of resources for neonates with HSV. The median hospital charge was US$37,431 (interquartile range US$14,667–US$74,559) per infant.^21^ The financial burden for congenital rubella syndrome is also substantial, estimated at US$4,200 to US$57,000 per case annually in middle-income countries and up to US$140,000 over a lifetime in high-income countries.^22^

Given the unfolding epidemiology, limited data, severe complications, and high economic burden of TORCH infections in the care of neonates with 1 or more of these infections, it is critical to thoroughly survey the distribution of TORCH infections in northern Tanzania. TORCH infections pose a substantial public health problem because the infected mothers are mostly asymptomatic, but the infections can lead to death, organ injury, or severe short- and long-term sequelae for their unborn fetuses and newborns. Thus, we aimed to study the immune status to congenital infections by TORCH agents among pregnant women and their newborns in northern Tanzania.

## METHODOLOGY

### Study Design

This was a cross-sectional study conducted from December 2013 to April 2014 at the Kilimanjaro Christian Medical Centre (KCMC), in Moshi, northern Tanzania. The study was conducted in the Obstetrics and Gynaecology Department and Paediatrics and Child Health Department using the antenatal clinic, labour ward, and neonatal ward. KCMC is a faith-based institution, primarily serving patients from the Kilimanjaro Region and neighbouring regions, such as Arusha, Manyara, Singida, and Tanga, as well as other parts of Tanzania. The number of hospital deliveries range from 3,500 to 4,000 per year, with more than half from the Moshi urban area and nearly 20% referred for medical reasons.^23^

### Study Population

**FIGURE 1.**
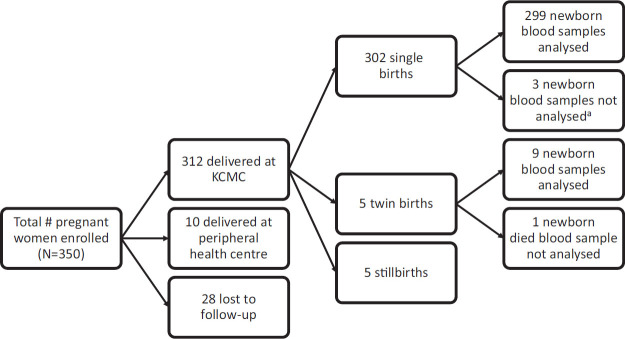
Number of Participants Recruited and Newborn Blood Samples Analysed

Abbreviations: KCMC, Kilimanjaro Christian Medical Centre.

^a^The sample size was not sufficient and therefore was not analysed.

All pregnant women above the age of 18 who attended the antenatal clinic during the study period, had a gestational age of at least 28 weeks (according to last menstrual period), and gave voluntary consent were enrolled in the study (n=350). Pregnant women not planning to deliver at KCMC and those with emergency referrals for delivery were excluded. Of the 350 pregnant women enrolled in the study, 312 delivered at KCMC, 10 delivered at nearby health centres, and 28 were lost to follow-up (Figure 1).

### Sample Size Estimations

The minimum sample size was estimated using a formula by the Survey System software package (1988), expressed as [Z^2^·(p)·(1 p)]/c^2^, where Z=1.96 for 95% confidence level (CI). A prevalence percentage (p) of 35% was selected based on a study done in Dar es Salaam^18^ and c represented the minimal tolerable error at 95% CI, expressed as a decimal (.05). The minimum estimated sample size was 344 participants. The study therefore recruited 350 pregnant women and their infants (mother-infant pairs).

### Data Collection

We collected sociodemographic, behavioural, clinical, and delivery data of mothers enrolled in the study using pre-tested data collection tools. Data collected included age, level of education, marital status, alcohol consumption, parity, mode of delivery, and related maternal and fetal outcomes.

After enrolment, a laboratory request form was administered. Approximately 4 ml of whole blood was collected from each participant using BD Vacutainer blood collection redtop tubes (BD Medical, Plymouth, UK). Blood was allowed to clot for 1 hour and transported to Kilimanjaro Christian Research Institute – a biotechnology laboratory situated within the KCMC campus. Centrifugation was done at 1000–1300 g for 10 minutes. The supernatant (ie, serum) was transferred into cryotubes and stored in a refrigerator at –70°C for later analysis. All newborns were followed up immediately after delivery and 2 ml of cord blood were collected in BD Vacutainer blood collection red-top tubes for both liveborn and stillborn babies. The blood samples were processed and stored as described earlier.

### Laboratory Analysis

Laboratory analysis was done at the Kilimanjaro Christian Research Institute. All blood samples and kit reagents were brought to room temperature (23°C–25°C) before sample analysis was done. This was followed by testing for antibodies (IgG and IgM) to toxoplasmosis, rubella, CMV, HSV-1, and HSV-2 using an enzyme-linked immunosor-bent assay (ELISA) test kit for mothers and only immunoglobulin M (IgM) antibodies to TORCH for newborns, according to the manufacturer's instructions, as used for serum testing in this study.

#### Assay Procedure for TORCH IgG

Ten microlitres (10 ll) of blood sample were diluted with 1 ml of sample diluent. For each standard, 100 μl of diluted test sera were added into microplate wells and covered with cardboard sealer. The microplate wells were incubated for 30 minutes at room temperature (22°C–28°C). The plate covers were removed and contents discarded. The microplate wells were washed 5 times (each with 300 lL of working wash solution). A 100 μl of conjugate solution was added into all wells and covered with cardboard sealer. The microplate wells were incubated for 30 minutes at room temperature (22°C–28°C). The plate covers were removed and contents discarded. The micro-plate wells were washed 5 times. Each well was mixed with 100 μl of chromogenic substrate solution and then incubated at room temperature in a dark room for 15 minutes. To stop the reaction, 100 μl of stop solution was added to the wells. Absorbance was recorded at 450 nm by an ELISA reader.

#### Assay Procedure for TORCH IgM

We added 100 μl of each control as well as diluted test sera into appropriate wells. Two consecutive wells in the first strip were considered blank and positive, respectively, and the next 2 wells for duplicate negative control serum. The microplate wells were covered with cardboard sealer tightly and incubated for 30 minutes at 37°C. The plate covers were removed and contents discarded. The micro-plate wells were washed 5 times, each with 300 μl of working wash solution. A 100 μl conjugate solution was then added into all wells except the blank well. The micro-plate wells were covered with cardboard sealer tightly and incubated for 30 minutes at 37°C. The plate covers were removed and contents discarded. The microplate wells were washed 5 times. Each well was mixed with 100 μl of chromogenic substrate solution and then incubated at room temperature in a dark room for 15 minutes. To stop the reaction, 100 μl of stop solution was added to the wells. Absorbance was recorded at 450 nm by an ELISA reader.

Validation of serological assays was done and the cut-off values for detection of IgG and IgM were calculated according to the manufacturer's instructions.

#### Result Calculation and Evaluation for TORCH IgG

To distinguish between positive and negative results, the serum/cut-off ratio (S/Co) index was used and calculated according to manufacturer's index cut-off value for this ELISA:

S/Co=sample optical density (OD)/cut-off value (Cut-off value=10 antibody units per ml standard mean OD)

Based on this index, results higher than 1.1 were considered positive and results less than 0.9 were considered negative.

#### Result Calculation and Evaluation for TORCH IgM

To distinguish between positive and negative results, the cutoff index was determined by using the following formula as per the manufacturer manual:

Cut-off index=OD of sample/cut-off value

(Cut-off value= mean OD of negative control serum + 0.15)

### Statistical Analysis

Data were analysed using IBM SPSS Statistics v. 20 (Armonk, New York, USA) where descriptive statistics were estimated. For combined TORCH infection, univariate analysis was done to estimate the odds ratios and the 95% confidence interval to determine factors associated with exposure to both TORCH infections with *P*<.05 used as a cut-off value to indicate statistical significance.

### Ethical Consideration

Ethical clearance was obtained from the Kilimanjaro Christian Medical University College via the College Research Ethics Review Committee and permission was obtained from KCMC Obstetrics and Gynaecology Department and Paediatrics and Child Health Department. Assent to participate and informed written consent was sought from each pregnant woman prior to involvement in the study.

## RESULTS

### Sociodemographic Characteristics of the Study Participants

The mean age of the pregnant women was 29 (standard deviation [SD]=5.1) years at enrolment, 191 participants (54.6%) were between 21 and 30 years old, and 201 (57.4%) participants were married. Most of the participants (74.9%) had attended secondary education and the majority of them were living in urban areas (89.1%). Table 1 shows the socio-demographic characteristics of the study participants.

**TABLE 1.** Sociodemographic Characteristics of the Participants (N=350)

Characteristics	n	%
Age in years		
≤ 20	15	4.2
21–30	191	54.6
31–40	142	40.6
41+	2	0.6
*Mean age in years (SD)*	*29.1 (5.1)*	
Marital status		
Single	2	0.6
Married	201	57.4
Cohabiting	147	42.0
Level of education		
Primary education	88	25.1
Secondary education and above	262	74.9
Occupational status		
Unemployed	3	0.9
Self-employed	144	41.5
Employed	144	41.5
Housewife	36	10.4
Student	20	5.8
Residence		
Rural	38	10.9
Urban	312	89.1

Abbreviation: SD, standard deviation.

### Behavioural, Clinical, and Delivery Characteristics of the Participants

About 15% of the study participants reported to have consumed alcohol during the current pregnancy (Table 2). A total of 12 (3.4%) pregnant women enrolled in this study were HIV positive. Fifty-nine percent tested negative for the venereal disease research laboratory syphilis test and 40.3% did not know their syphilis infection status. Regarding parity, 240 (68.6%) participants were multiparous. The mean gestational age was 38.2 weeks (SD=3.3). Regarding the mode of delivery, 187 (60.1%) participants underwent spontaneous vaginal delivery and single live birth was the most common delivery outcome (96.8%). No major post-delivery complications among mothers were reported. History of prolonged rupture of membrane was rare.

**TABLE 2.** Behavioural, Clinical, and Delivery Characteristics of Participants

Characteristics (n)	n	%
Drug abuse or addiction (350)		
Alcohol	54	15.4
None	296	84.6
HIV status (350)		
Positive	12	3.4
Negative	331	94.6
Unknown	7	2.0
Syphilis status (350)		
Positive	2	0.6
Negative	207	59.1
Unknown	141	40.3
Parity (350)		
Nulliparous	110	31.4
Multiparous	240	68.6
*Mean age in years at gestation (SD)*	*34.2 (3.3)*	
Place of delivery (350)		
Kilimanjaro Christian Medical Centre	312	89.0
Peripheral health centre	10	2.9
Lost on follow-up	28	8.0
Mode of delivery (311)		
Spontaneous vaginal delivery	187	60.1
Caesarean section	124	39.9
Pregnancy outcomes (311)		
Singleton live birth	301	96.8
Multiple live births	5	1.6
Stillbirth, macerated	2	0.6
Stillbirth, fresh	3	1.0

Abbreviation: SD, standard deviation.

### Seroprevalence of IgG and IgM Antibodies to TORCH in Pregnant Women

Overall, 141 (40.6%) pregnant women participants were seropositive for all 4 TORCH infections in our study. Ages above 35 years (OR 6.15; 95% CI, 1.22–31.19; P=.028) and multiparity (OR 1.63; 95% CI, 1.01–2.62; P=.045) were associated with a higher risk of seropositivity for all TORCH infections (Table 3).

**TABLE 3.** Maternal and Clinical Characteristics of Participants with IgG Antibodies to TORCH Infections Present (N=347)

		All TORCH IgG		
Characteristics	Total	n (%)	OR (95% CI)	*P*-value
Age (years)				
≤ 20	15	2 (13.2)	Ref.	
21–35	295	121 (41.0)	4.52 (1.00–20.39)	.050
36+	37	18 (48.6)	6.15 (1.22–31.19)	.028
Marital status				
Single	2	1 (50.0)	1.81 (0.11–29.50)	.678
Married	199	88 (44.2)	1.43 (0.92–2.22)	.108
Cohabiting	146	52 (35.6)	Ref.	
Level of education				
Primary	86	36 (41.9)	1.07 (0.65–1.75)	.789
Secondary and above	261	105 (40.0)	Ref.	
Occupational status				
Unemployed	3	1 (33.3)	1.30 (0.11–16.0)	.838
Self-employed	144	67 (46.5)	2.26 (1.02–5.03)	.045
Employed	144	57 (39.6)	1.70 (0.76–3.80)	.193
Housewife	36	10 (27.8)	Ref.	
Student	20	6 (30.0)	1.11 (0.34–3.71)	.860
Residence				
Rural	36	14 (38.9)	Ref.	
Urban	311	127 (40.8)	1.09 (0.54–2.20)	.822
Parity				
Nulliparous	107	35 (32.7)	Ref.	
Multiparous	240	106 (44.2)	1.63 (1.01–2.62)	.045
History of PROM				
Yes	10	3 (30.0)	Ref.	
No	298	122 (40.9)	1.62 (0.41–6.38)	.365
Mode of delivery				
SVD	188	71 (37.8)	Ref.	
C/S	122	56 (45.9)	1.40 (0.88–2.22)	.155
HIV status				
Positive	12	7 (58.3)	2.05 (0.64–6.60)	.228
Negative	328	133 (40.5)	Ref.	
Unknown	7	1 (14.3)	0.24 (1.03–2.05)	.194
Syphilis status				
Positive	2	2 (100.0)	2.86 (0.000)	.999
Negative	205	74 (36.1)	Ref.	
Unknown	140	65 (46.1)	1.53 (0.99–2.38)	.055

Abbreviations: C/S, caesarean section; PROM, premature rupture of membrane; Ref., reference group; SVD, spontaneous vaginal delivery.

**FIGURE 2.**
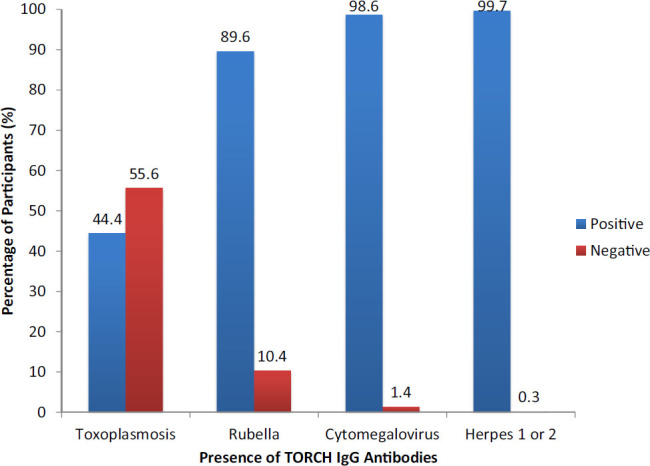
Prevalence of TORCH IgG Antibodies Among Participants, by Co-Infection

**FIGURE 3.**
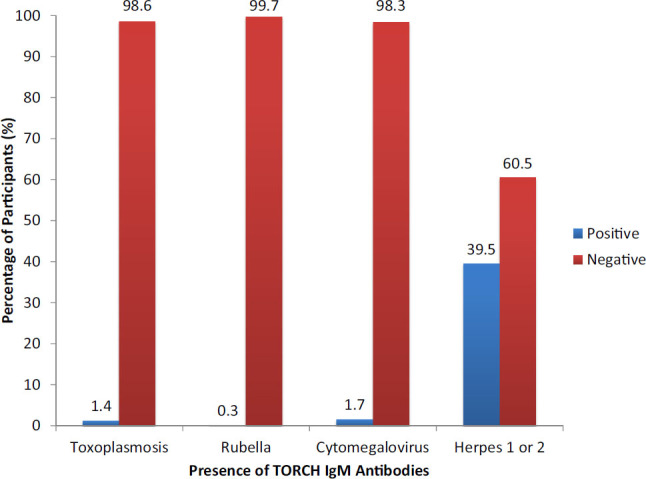
Prevalence of TORCH IgM Antibodies Among Participants, by Co-infection

Seroprevalence of IgG antibodies was as follows: toxoplasmosis 154 (44.4%), rubella 311 (89.6%), CMV 342 (98.6%), and HSV-1 and HSV-2 (99.7%) (Figure 2). Seroprevalence of IgM antibodies to HSV-1 and HSV-2 was 137 (39.5%) (Figure 3).

### Seroprevalence (IgM) Antibodies to TORCH in Newborns

Seroprevalence of IgM antibodies to HSV-1 and HSV-2 among newborns was 11 (3.6%). One newborn was coinfected with rubella, for a prevalence of 0.3% for rubella.

**FIGURE 4.**
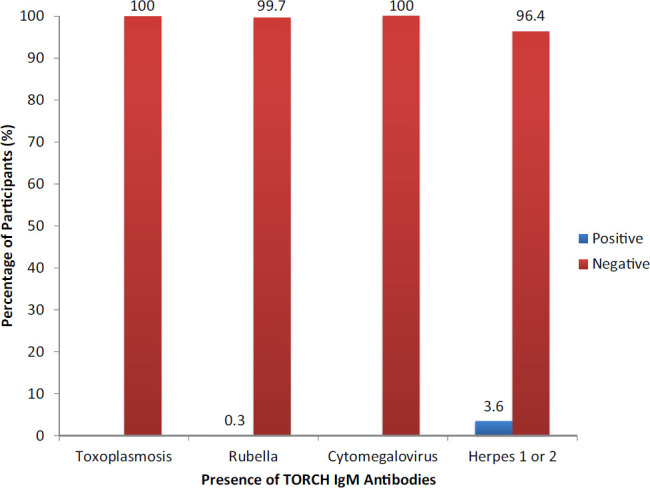
Prevalence of TORCH IgM Antibodies Among Newborns, by Co-Infection

The mothers of 9 of the 11 newborns had previously been exposed to all TORCH infections investigated in this study: toxoplasmosis, rubella, CMV, and HSV-1 and HSV-2. All newborns who tested positive for IgM antibodies were single births, born with Apgar scores above 7, had no reported birth trauma or birth defects, had HIV-negative mothers, lived in urban areas, and their mothers had no history of premature rupture of membranes during labour. None of the newborns were infected with toxoplasmosis or CMV (Figure 4).

## DISCUSSION

The objective of this study was to investigate previous exposure to TORCH infections among pregnant women and their newborns receiving care at KCMC in Moshi, Tanzania, by measuring their levels of antibodies to the infections. Our findings showed a high seroprevalence of IgG antibodies to toxoplasmosis, rubella, CMV, and HSV-1 and HSV-2. A high seroprevalence of IgM antibodies to HSV may indicate ongoing infection, which increases risk of HSV transmission to the newborn. We also found that 3.6% of the newborns born to mothers with HSV tested positive for IgM antibodies, which indicates maternal intrauterine transmission. Overall, 40.6% of pregnant women were seropositive for all TORCH infections in this study. Higher exposure to these infections may therefore lead to concurrent infection, which could increase the chances of transmission, as observed in 9 of 11 newborns in this study who were HSV-positive and born to mothers who tested positive for IgG antibodies to all TORCH infections in this study. The higher IgG seroprevalence observed in this study in – which nearly all women were previously exposed to some TORCH infections – may indicate that women of reproductive age are exposed to the infections before pregnancy in the general population. This may support generalizability of the study findings; however, because it was a highly selective population of study participants in a tertiary care hospital, we must be cautious about generalizing the findings.

Previous exposure to toxoplasmosis among pregnant women in our study was high, which could be attributed to geographical conditions, personal hygiene, eating habits, and lack of awareness of the disease before conception. Similar findings of seroprevalence for toxoplasmosis were reported in Colombia (45.8%)^24^ and Albania (48.6%).^25^ On the other hand, our findings showed a higher sero-prevalence for TORCH infections than in Mexico (8.2%),^26^ the United Kingdom (9.1%),^27^ Japan (10.3%),^28^ Burkina Faso (20.3%),^29^ Palestine (27.9%),^30^ Croatia (29.1%),^31^ Kosovo (29.4%),^32^ Nigeria (32.6%),^33^ and Iran (37.2%).^34^ More-over, our findings showed a higher seroprevalence than recently described by Mwambe and colleagues in Tanzania (30.9%).^6^ Comparatively, our findings show a lower prevalence than reported in Saudi Arabia (51.4%)^35^ and some parts of Brazil (59.8% in Palotina and 60.6% among Jesuits).^36^ The variation of prevalence in different countries and regions may be attributed to geographical conditions, personal hygiene, eating habits, and lack of awareness of the disease. In our study, no newborn tested seropositive for antibodies to toxoplasmosis. The majority of newborns infected during pregnancy are asymptomatic at birth; however, 80% of them may develop long-term learning and visual disabilities later in their lives.^37^

Exposure to rubella before conception among pregnant women in this study was high, which may indicate a heavy circulation of wild-type rubella viruses in our population because widespread vaccination is not yet available in Tanzania.^38^ Moreover, infection of rubella virus leads to lifelong immunity. Similar trends of exposure to rubella were reported in Nigeria (87.5%),^39^ Tanzania (92.9%),^40^ Haiti (93.4%),^41^ Turkey (93.8%),^42^ and Croatia (94.6%)^31^; however, our findings showed a higher prevalence to rubella than reported in Burkina Faso (77%)^43^ and western Sudan (65.3%).^44^

Exposure to CMV among pregnant women was also high in our study. The risk of CMV exposure to women of reproductive age increases when their child attends daycare^12^ and the fact that the majority of participants in our study were educated, employed, multiparous, and lived in urban areas, where the possibility of children attending daycare is high, may contribute to the high levels of exposure to CMV. Similarly high exposure rates were reported in Iran (97.7%),^45^ Nigeria (97.2%),^46^ Palestine (96.6%),^47^ and Taiwan (91.1%)^48^; however, lower rates of exposure were reported in Croatia (75.4%),^31^ Norway (62.8%),^49^ and Tanzania (63.1%) from a study in 1990.^50^

Additionally, exposure to HSV-1 and HSV-2 among pregnant women in the study was high. This could be explained by the changing epidemiology of HSV disease toward HSV type-1 more than type-2, changing sexual behaviour, and the latency of HSV post-primary infection. Similar findings have been reported in the Republic of Vanuatu (100%)^51^ and Croatia (85.5%)^32^; however, lower exposures were reported in Norway (14%),^18^ Belgium (18.2%),^52^ and in the Manyara and Singida regions of Tanzania (20.7%).^17^ Parallel findings were seen among women with high risk reported in northern Tanzania.^16^ The risk of transmission of HSV-1 and HSV-2 to newborns was only from mothers with antibodies (ie, IgG and IgM antibodies to HSV-1 and HSV-2). This may be explained by acute maternal infection or reactivation of latent infection.

HSV was the only one with higher transmission rate (3.6%), however, newborns were asymptomatic at birth and during the early neonatal period. A review of congenital herpes cases in the past 4 decades revealed that among 64 cases with clinical presentations, cutaneous lesions were the most common clinical manifestation where less than one-third of the cutaneous presentation had typical triad findings at birth, 44% had manifestations other than vesicles or bullae, 67% of patients had central nervous system manifestation and 39% patients had ocular findings, 18% of whom had retinal disease.^53^ Neonatal HSV infection is treated with acyclovir, 60 mg/kg of body weight per day in 3 doses (20 mg/kg per dose) given intravenously. Disseminated and central nervous system infections are treated for at least 21 days.^20^ Currently, no treatment protocol exists at KCMC for newborns with HSV infection; therefore, no treatment is given for newborns who test HSV positive.

### Strengths and Limitations

This study had a large sample size that included both pregnant women and their newborns. We were therefore able to estimate antibodies for 4 infections belonging to the TORCH group of infections and the transmission rate, primarily for HSV-1and HSV-2.

Despite its strengths, this study also had some limitations. Limitations included a highly selective population of study participants because it was a hospital-based study in a tertiary care hospital, and therefore our findings might not reflect exposure in the general population. Second, the loss to follow-up of nearly 10% of newborns in our study means that we do not know their serostatus.

## CONCLUSIONS AND RECOMMENDATIONS

Exposure to TORCH infections was very high among pregnant women in our study population. Newborn seroprevalence of IgM antibodies to HSV-1 and HSV-2 was also high. HSV-1 and HSV-2 infections in pregnant women may disturb maternal, foetal, and neonatal health, and therefore, antenatal screening may be recommended. A large cohort study could help provide the evidence needed, including longterm sequelae for newborns who test positive for TORCH antibodies, in order to advocate for TORCH management during pregnancy and newborn care at KCMC.

HSV prevention advocacy to both partners during antenatal care is also recommended. Higher IgM antibodies to HSV-1 and HSV-2 among pregnant women and their newborns indicate a need to establish treatment protocol to support management of pregnant women and newborns who are seropositive for IgM antibodies.
